# Functional and morphological renal changes in a Göttingen Minipig model of obesity-related and diabetic nephropathy

**DOI:** 10.1038/s41598-023-32674-6

**Published:** 2023-04-12

**Authors:** Berit Østergaard Christoffersen, Camilla Aarup Kristensen, Rikke Lindgaard, Rikke Kaae Kirk, Birgitte Martine Viuff, Peter Helding Kvist, Henrik Duelund Pedersen, Trine Pagh Ludvigsen, Tine Skovgaard, Johannes Josef Fels, Torben Martinussen, Liselotte Bruun Christiansen, Susanna Cirera, Lisbeth Høier Olsen

**Affiliations:** 1 Novo Nordisk A/S, Måløv, Denmark; 2 Department of Veterinary and Animal Sciences, University of Copenhagen, Frederiksberg, Denmark; 3 Department of Public Health, Faculty of Health and Medical Sciences, University of Copenhagen, Copenhagen, Denmark; 4 Present Address: AJ Vaccines A/S, Copenhagen S, Denmark; 5 Present Address: AniCura ApS, Herlev, Denmark; 6 Present Address: Danish Medicines Agency, Copenhagen, Denmark; 7 Present Address: Unilabs, Copenhagen, Denmark; 8 Present Address: Novo Nordisk A/S, Søborg, Denmark

**Keywords:** Endocrinology, Nephrology, Pathogenesis, Diseases, Endocrine system and metabolic diseases, Kidney diseases

## Abstract

Obesity-related glomerulopathy and diabetic nephropathy (DN) are serious complications to metabolic syndrome and diabetes. The purpose was to study effects of a fat, fructose and cholesterol-rich (FFC) diet with and without salt in order to induce hypertension on kidney function and morphology in Göttingen Minipigs with and without diabetes. Male Göttingen Minipigs were divided into 4 groups: SD (standard diet, n = 8), FFC (FFC diet, n = 16), FFC-DIA (FFC diet + diabetes, n = 14), FFC-DIA + S (FFC diet with extra salt + diabetes, n = 14). Blood and urine biomarkers, glomerular filtration rate (GFR), blood pressure (BP) and resistive index (RI) were evaluated after 6–7 months (T1) and 12–13 months (T2). Histology, electron microscopy and gene expression (excluding FFC-DIA + S) were evaluated at T2. All groups fed FFC-diet displayed obesity, increased GFR and RI, glomerulomegaly, mesangial expansion (ME) and glomerular basement membrane (GBM) thickening. Diabetes on top of FFC diet led to increased plasma glucose and urea and proteinuria and tended to exacerbate the glomerulomegaly, ME and GBM thickening. Four genes (CDKN1A, NPHS2, ACE, SLC2A1) were significantly deregulated in FFC and/or FFC-DIA compared to SD. No effects on BP were observed. Göttingen Minipigs fed FFC diet displayed some of the renal early changes seen in human obesity. Presence of diabetes on top of FFC diet exacerbated the findings and lead to changes resembling the early phases of human DN.

## Introduction

Diabetic nephropathy (DN) is one of the most serious chronic complications of diabetes and the condition is associated with considerable risk of morbidity and mortality. DN is the leading cause of end-stage renal disease and has a prevalence of up to 30–40% of diabetic patients^1–3^. In addition, obesity-related glomerulopathy (ORG) is observed with increasing frequency in non-diabetic people living with obesity^4^. The two diseases share many of the same renal morphological and functional changes and the risk factors for disease development are overlapping^1,5^, indicating a common early pathogenesis.

The complex and multifactorial pathogenesis of both ORG and DN remains to be fully elucidated but involves both metabolic and haemodynamic disturbances, and is associated with risk factors such as obesity, insulin resistance, hyperglycemia, dyslipidemia and hypertension^1,5^. The two diseases typically evolve over several years, and there is an unmet need for therapies that halt the progression of the early changes into more advanced disease.

It is not possible to diagnose or monitor early stages of ORG and DN by using the circulating biomarkers traditionally used in kidney disease, i.e. blood urea nitrogen and creatinine or creatinine-derived glomerular filtration rate (GFR), since these biomarkers are not specific, do not reflect small or early changes in renal function, and can only be considered reliable biomarkers in the late stages when significant impairment of the renal function is present^6,7^. Also, microalbuminuria, one of the hallmarks of DN, typically is not present in the very early disease stages and is not a sensitive predictor of disease progression^8^.

Of the newer biomarkers for DN, the tubulo-interstitial biomarker neutrophil gelatinase-associated lipocalin (NGAL) has been shown to be increased in urine prior to the occurrence of microalbuminuria and may serve as a complementary prognostic marker^6,9^. Also, the imaging biomarker resistive index (RI) assessed by Doppler ultrasound has been shown to be increased in obesity^10^ and in the early stages of DN before GFR declines are observed^11.^

From an animal model perspective, benefits may be gained from supplementing the existing rodent models with a porcine model, since the pig is more human-like on several key parameters such as the structure and function of the kidney^12,13^, the inflammasome^14^, and the metabolism and plasma lipid profile^15^. Minipigs are especially attractive to use in long-term studies due to their small size, and studies in minipigs, indicate that early-stage nephropathy can be induced by long-term hyperglycemia, Western diets or a combination of the two^16–18^. More late-stage models with an accelerated disease development would be attractive, and this may be achieved by combining hyperglycemia with some of the other risk factors for developing diabetic nephropathy, such as diet-induced obesity/metabolic syndrome and hypertension. In the present study, obesity and metabolic syndrome were induced by feeding a fat, fructose and cholesterol-rich (FFC) diet, while hypertension was theoretically induced by including a group with a high dietary salt content.

The aim of this study was thus to evaluate changes in functional and morphological renal parameters in Göttingen Minipigs fed an FFC diet with or without chemically induced diabetes and with or without extra dietary salt/hypertension. The evaluations included plasma and urinary biomarkers relevant for ORG/DN, GFR evaluated by inulin clearance, blood pressure (BP), RI, gene expression analyses and histopathological and electron-microscopical examinations of renal tissue samples.

## Methods

### Animals, diet and housing

Fifty-two castrated male Göttingen Minipigs (Ellegaard Göttingen Minipigs A/S, Dalmose, Denmark) aged approx. seven months at study start were included in the study. The average body weight was 16 ± 2 kg. The animals were part of a larger study (n = 84 in total) focusing on other end points (see Supplementary information file 1).

**Figure 1:**
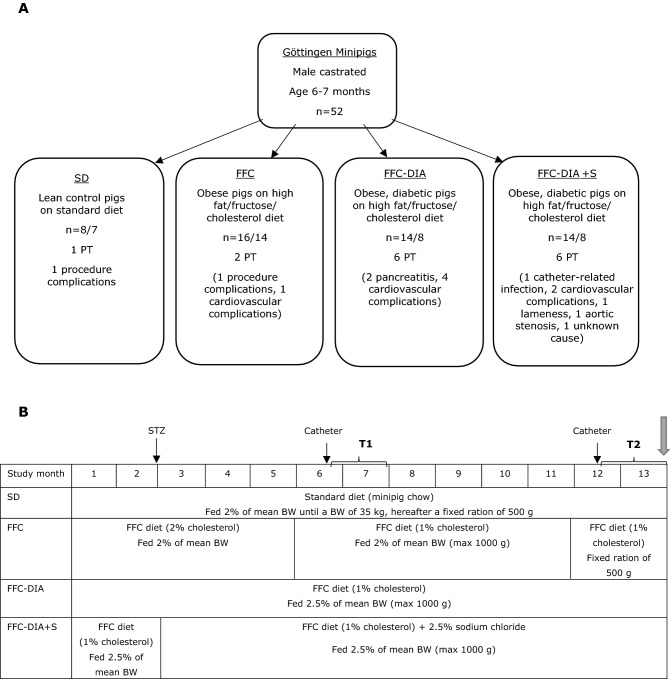
Study overview. (**A**) Fifty-two male castrated Göttingen Minipigs were included in the study and 15 were terminated prematurely. The numbers and reasons for premature termination (PT) are described under each group. The procedure-related complications occurred during investigations of other end points in the same animals (unrelated to the present study). (**B**) Timeline and details of feeding and tests in each of the four groups over the study period. FFC: high fat/fructose/cholesterol diet, STZ: induction of diabetes with streptozotocin, T1: mid-study in vivo evaluations, T2: end-study in vivo evaluations. Grey arrow indicates termination and tissue sampling. Modified from Schumacher et al. with permission^50^.

The study was run in 3 staggered cohorts, with animals from all groups equally represented in all cohorts, and animals from each group represented in random order on each study day. After an acclimation period of 2 weeks, the animals were weight-stratified into 3 groups according to diet and diabetic state: SD (fed standard diet, Mini-Pig, SDS, UK) (n = 8), FFC (fed high fat/fructose/cholesterol diet with 2% cholesterol (5B4L) for the first 5 months of the study, then changed to same diet with 1% cholesterol (9G4U) for the rest of the study) (n = 16) and FFC-DIA/FFC-DIA + S (fed FFC 1% cholesterol diet until chemical diabetes induction in week 9, n = 28). After diabetes induction, these animals were allocated into two groups based on their blood sugar levels (aiming at similar blood sugar levels in the two diabetic groups): FFC-DIA (continued on FFC 1% cholesterol diet for the rest of the study) (n = 14) and FFC-DIA + S (switched to FFC 1% cholesterol diet with extra salt (5BTJ, sodium chloride content 2.5%) after diabetes induction) (n = 14) (all FFC diets from TestDiet®, Missouri, US) (Fig. 1). Nutritional compositions of the diets can be found in Supplementary information file 2. The animals were fed once daily and had free access to drinking water; straw was used as bedding and rooting material and apples were given as treats. The animals were housed at the research facilities at the University of Copenhagen and were monitored daily during caretaking and dosing of insulin. They were group housed except for periods with intravenous (IV) catheters implanted and/or wearing blood pressure telemetry equipment.

### Study overview, in vivo evaluations and tissue sampling

In vivo evaluations were performed at mid study (T1, from 6–7 months after study start) and repeated at end study (T2, from 12–13 months after study start) (Fig. 1B). In vivo evaluations included weighing, measurement of body composition by dual energy x-ray absorptiometry (DEXA) scanning, blood and urine sampling, GFR measurement by inulin clearance test, BP measurements by telemetry, and RI assessment by ultrasound scanning. All in vivo methods are described in detail in Supplementary information file 3.

Study duration was 13 months and at the end of the study the animals were euthanised by exsanguination during deep surgical anaesthesia using tiletamin and zolazepam mixture (Zoletil 50 Vet, ChemVet, Silkeborg, Denmark) with added ketamine (Ketaminol Vet (100 mg/mL) Intervet, Skovlunde, Denmark), xylazine (0.84 mg/kg) (Rompun Vet (20 mg/mL) Bayer, Lyngby, Denmark) and buthorphanol (0.16 mg/kg) (Torbugesic (10 mg/mL) Scanvet, Fredensborg, Denmark), after which the kidneys were harvested for histopathological and electron microscopical evaluations and gene expression analysis as described in Supplementary information file 3. All tissue analyses were blinded (investigators were unaware of group allocations).

### Catheter implantation

A central-venous catheter was implanted under general anesthesia at least 1 week before procedures requiring venous access (diabetes induction or test periods) using one of the 3 methods described in Supplementary information file 3. The catheter was used for diabetes induction, stress-free blood sampling and infusion of glucose and inulin during tests.

### Induction of diabetes

Six to eight weeks into the study, after 4–6 weeks on the experimental diets, a type 1-like (insulin-dependent) diabetes was induced in 28 animals (FFC-DIA and FFC-DIA + S) by streptozotocin (STZ, Sigma-Aldrich, Denmark), 60 mg/kg given intravenously (IV) once daily on three consecutive days (protocol modified from Gerrity et al.^19^). Fasting blood glucose level was measured daily for the first 8 days after induction and then 1–2 times weekly (glucometer Accu-Chek® Aviva, Roche Diabetes Care, Roche Danmark). Daily subcutaneous injections with insulin glargine (Lantus®, Sanofi-Aventis, Frankfurt am Main, Germany) were given in the morning in relation to the meal to keep the fasting morning blood glucose at approx. 15 mM. Approximately three weeks after diabetes induction, the animals were allocated into two diabetic groups FFC-DIA (n = 14) and FFC-DIA + S (n = 14) with similar mean plasma glucose level.

### Serum, plasma and urine analysis

Serum, plasma and urine analyses were performed as described in Supplementary information file 3.

### Calculations and statistical analyses

Data are presented as median and interquartile range (Q1-Q3). . GFR (inulin curves used for calculation are presented in Supplementary information file 4) was given as absolute value (ml/min/pig). Albumin, protein and NGAL concentrations in urine were corrected for urine creatinine concentrations, giving the following derived parameters: uACR (urine albumin/urine creatinine), uPCR (urine protein/urine creatinine) and uNGALCR (urine NGAL/urine creatinine).

Statistical analysis, Spearman correlation analysis and principal component analysis (PCA) were performed using SAS version 9.4, SAS Studio (Statistical Analysis Software, SAS Institute Inc., Cary, NC, USA) or RStudio (gene expression analyses, Rstudio Team, 2015). Graphs were made with GraphPad Prism (GraphPad Software, La Jolla, CA, USA). No power calculations were performed, since no a priori knowledge of effect size and variation for the renal end points was available. Further details can be found in Supplementary information file 3.

### Ethical approval

The study was approved by the Animal Experiments Inspectorate, Ministry of Food, Agriculture and Fisheries, Denmark (License No. 2012-15-2934-00715). All procedures were performed in accordance with the EU directive on protection of Animals for Research Purposes Directive 2010/63/EU. The study is reported in accordance with the ARRIVE guidelines.

## Results

Thirty-seven animals out of 52 completed the study (see Fig. 1 for causes of premature termination). The key data and statistics are presented in Tables and Supplementary information file 5.

### BW and body composition

Body weight and Fat% were significantly higher in the three FFC-groups compared to SD at both T1 and T2. At T2, the FFC group had higher Fat% than FFC-DIA + S whereas no significant differences were observed between the two FFC-DIA groups. Both BW and Fat% increased significantly from T1 to T2 (Table 1).

**Table 1:** Basic characteristics of castrated male Göttingen Minipigs fed with standard diet (SD) or fat, fructose and cholesterol rich diet (FFC) with or without additional salt (S) and with or without streptozotocin-induced diabetes (DIA).

Parameter	Study period	SD n = 8	FFC n = 16	FFC-DIA n = 14	FFC-DIA + S n = 14	Overall significant *P*-values
Bodyweight (kg)	T1	29.5 (28.0–30.5)	43.5 (40.0–47.5)	35.5 (32.0–38.0)^12^	36.0 (32.0–39.0)^9^	^#^Group*time (*P* = 0.0011): FFC > FFC-DIA, FFC-DIA + S > SD T2 > T1
	T2	39.0 (38.0–41.0)^7^	78.0 (69.0–81.0)^14^	59.5 (54.8–64.0)^8^	53.5 (49.0–68.0)^8^	
Fat free body mass (FFBM) (kg)	T1	22.4 (20.9–23.0)	27.1 (25.0–28.2)^14^	21.6 (20.1–23.8)^8^	22.6 (21.6–23.9)^8^	Group*time (*P* < 0.0001): T1: FFC > SD, FFC-DIA, FFC-DIA + S T2 > T1: SD, FFC-DIA, FFC-DIA + S
	T2	26.9 (24.0–28.6)^7^	27.0 (24.8–27.8)^14^	25.4 (22.9–27.0)^8^	24.9 (23.2–29.6)^8^	
Total Body Fat (%)	T1	21.5 (19.5–28.2)	40.2 (37.6–41.9)^14^	36.5 (33.1–38.0)^12^	34.8 (28.5–37.6)^9^	^#^Group*time (*P* = 0.0009): T1 and T2: FFC, FFC-DIA, FFC-DIA + S > SD T2: FFC > FFC-DIA + S T2 > T1
	T2	27.6 (24.0–30.7)^7^	64.2 (61.4–67.6)^14^	54.8 (52.7–56.0)^8^	53.1 (47.9–61.0)^8^	
Systolic BP (mmHg)	T1	131 (128–134)^7^	123 (111–129)^14^	116 (113–126)^10^	127 (115–134)^8^	Time (*P* = 0.0001): T2 > T1 Period (P < 0.0001): Day > night
	T2	137 (129–140)^6^	140 (132–154)^9^	127 (116–146)^7^	132 (113–141)^7^	
Diastolic BP (mmHg)	T1	95 (91–98)^7^	89 (84–94)^14^	88 (86–92)^10^	91 (87–101)^8^	^##^Time(*P* < 0.0004): T2 > T1 Period: (*P* < 0.0001): Day > night
	T2	97 (96–105)^6^	103 (101–111)^9^	96 (89–111)^7^	94 (86–101)^7^	
Mean BP (mmHg)	T1	115 (110–116)^7^	108 (98–113)^14^	103 (101–111)^10^	111 (103–118)^8^	Time (*P* < 0.0004): T2 > T1 Period: (*P* < 0.0001): Day > night
	T2	117 (113–122)^6^	123 (118–134)^9^	112 (104–130)^7^	115 (100–123)^7^	
Mean HR (beat/min)	T1	80 (76–84)^7^	94 (88–96)^14^	100 (97–104)^10^	98 (93–102)^8^	^###^Time (*P* < 0.0001): T1 > T2 Group (*P* < 0.0001): FFC-DIA, FFC-DIA + S > SD, FFC
	T2	72 (69–80)^6^	72 (71–75)^9^	96 (83–102)^7^	86 (76–105)^7^	
Circulating metabolic biomarkers						
Plasma glucose (mmol/L)	T1	3.7 (3.5–4.0)	3.8 (3.7–4.1)	14.9 (9.3–17.6)^12^	16.0 (10.4–17.0)^9^	^#^Group (*P* < 0.0001): FFC-DIA, FFC-DIA + S > SD, FFC
	T2	3.6 (3.3–3.9)^7^	3.7 (3.6–3.8)^13^	15.4 (14.7–19.0)^8^	13.7 (13.0–15.6)^7^	
Plasma fructosamine (µmol/L)	T1	250 (239–276)	239 (219–259)	479 (306–587)^12^	504 (478–562)^9^	^#^Group (*P* < 0.0001): FFC-DIA, FFC-DIA + S > SD, FFC
	T2	245 (214–271)^7^	240 (235–254)^13^	525 (450–560)^8^	480 (443–499)^7^	
Plasma triglycerides (mmol/L)	T1	0.34 (0.30–0.41)	0.57 (0.39–0.80)	0.87 (0.66–2.20)^12^	0.96 (0.69–1.41)^9^	^####^Group (T1: *P* = 0.0011, T2: *P* = 0.0006): T1: FFC-DIA, FFC-DIA + S,FFC > SD T2: FFC, FFC-DIA > SD
	T2	0.34 (0.29–0.35)^7^	0.63 (0.54–0.88)^13^	1.45 (0.83–2.00)^8^	1.13 (0.95–1.61)^7^	
Plasma total cholesterol (mmol/L)	T1	2.0 (1.7–2.2)	16.9 (11.4–21.3)	21.4 (10.6–32.4)^12^	14.6 (7.5–24.5)^9^	^####^Group (*P* = 0.0002): FFC, FFC-DIA, FFC-DIA + S > SD
	T2	1.7 (1.6–2.2)^7^	11.9 (11.0–13.2)^13^	18.9 (15.9–25.4)^8^	19.2 (11.3–26.3)^7^	
Plasma insulin (pM)	T1	20.4 (16.9–37.9)	36.8 (27.9–54.7)	8.5 (3.2–16.2)^12^	12.0 (3.0–19.3)^9^	^#^Group (*P* < 0.0001): SD, FFC > FFC-DIA, FFC-DIA + S
	T2	27.1 (16.9–43.6)^7^	55.5 (43.8–67.0)^14^	12.7 (5.7–15.4)^9^	5.4 (3.0–10.4)^8^	
Plasma glucagon (pM)	T1	7.7 (5.4–9.3)	16.0 (15.0–17.3)	29.9 (22.8–41.1)^12^	32.7 (20.2–36.9)^9^	^#^Group (*P* < 0.0001): FFC-DIA, FFC-DIA + S > FFC > SD Time (*P* = 0.0017): (T1 > T2)
	T2	6.7 (4.7–12.6)^7^	11.3 (8.9–13.8)^14^	25.9 (16.9–32.6)^9^	18.5 (12.9–22.4)^7^	

BP: Blood pressure, HR: Heart rate. Selected parameters were influenced by cohort: Body weight (CoA > CoB)(*P* = 0.024) and in addition plasma glucose (*P* = 0.0072 (CoA > CoC)), triglycerides at T1 (*P* = 0.048; CoA > CoC) ) and total cholesterol atT1 (*P* = 0.010; CoA > CoC) and* P* = 0.029; CoB > CoC).

T1: Approx. 7 months of diet-feeding, T2: Approx. 13 months of diet feeding.

Median and interquartile range (Q1-Q3). *P*-values < 0.05 were considered significant.

Superscript: Actual numbers included in the statistical analysis due to missing data. P-values from post hoc tests were adjusted using Bonferroni correction.

^#^Logaritmic transformation, ^##^Square root transformation, ^###^Logaritmic transformation: log(HRt1/1-HRt1), where HRt1 = (mean HR-50)/(164–50), ^####^Non parametric test.

### Circulating and urinary biomarkers

As expected, plasma glucose and fructosamine were significantly increased and insulin significantly decreased in the two diabetic groups compared to both the FFC and SD groups, and at both T1 and T2. Total cholesterol (TC) was significantly higher in all three FFC-groups compared to SD at both time points. Triglyceride (TG), urea and glucagon were significantly higher in the two diabetic groups compared to both FFC and SD. Furthermore, TG and glucagon were significantly higher in the FFC group compared to SD. Plasma creatinine was significantly lower in the two diabetic groups compared to both FFC and SD, and in the FFC group compared to SD at both T1 and T2. For plasma creatinine, the values were overall higher at T2 compared to T1, and for glucagon the values were higher at T1 compared to T2. No significant differences were observed in plasma NGAL concentrations at either time point.

At both T1 and T2, UPCR was higher in FFC-DIA compared to the three other groups, whereas no significant differences between the groups were found in UACR. UNGALCR was higher in the two diabetic groups compared to SD and FFC at both time points (Table 2).

**Table 2:** Kidney urine and circulating biomarkers in castrated male Göttingen Minipigs fed with standard diet (SD) or fat, fructose and cholesterol rich diet (FFC) with or without additional salt (S) and with or without streptozotocin-induced diabetes (DIA).

Parameter	Time	SD n = 8	FFC n = 16	FFC-DIA n = 14	FFC-DIA + S n = 14	Overall significant *P*-values
Circulating kidney biomarkers						
Plasma creatinine (mmol/L)	T1	0.073 (0.068–0.076)	0.055 (0.048–0.064)	0.036 (0.028–0.049)^12^	0.038 (0.026–0.050)	Group (*P* < 0.0001): SD > FFC > FFC-DIA, FFC-DIA + S Time (*P* < 0.0001): T2 > T1
	T2	0.082 (0.070–0.091)^7^	0.070 (0.058–0.077)^13^	0.039 (0.035–0.049)^8^	0.046 (0.041–0.067)^7^	
Plasma urea (mmol/L)	T1	1.9 (1.7–2.9)	2.9 (2.6–3.5)	3.4 (2.4–4.4)^12^	3.6 (2.9–3.8)	Group (*P* < 0.0001): FFC-DIA, FFC-DIA + S > SD, FFC
	T2	1.7 (1.6–2.0)^7^	2.4 (2.1–2.5)^13^	3.4 (2.8–3.8)^8^	3.9 (3.4–4.1)^7^	
Plasma NGAL (ng/mL)	T1	80.5 (70.0–95.6)^7^	74.9 (68.1–88.9)^14^	78.7 (67.5–82.1)^7^	73.8 (64.8–97.8)^8^	^#^NS
	T2	75.7 (63.6–91.2)^7^	77.0 (73.1–79.4)^14^	68.6 (51.5–76.4)^8^	79.8 (62.0–93.9)^8^	
Urinary kidney biomarkers						
Urinary glucose (mmol/L)	T1	0.92 (0.65–5.15)	1.00 (0.49–10.92)	64.6 (63.5–66.7)^12^	66.4 (64.4–66.8)^9^	^#^Group (*P* < 0.0001): FFC-DIA, FFC-DIA + S > SD, FFC
	T2	0.50 (0.16–0.92)^7^	0.59 (0.42–1.00)^14^	279.7 (134.5–361.9)^8^	147.0 (98.9–335.0)^9^	
UACR (mg/g)	T1	14.1 (3.65–47.8)	14.8 (5.65–52.25)	84.5 (26.2–270.2)^12^	18.4 (4.2–36.6)^9^	^##^T1:Group (P = 0.047) Posthoc tests NS
	T2	8.2 (4.3–9.8)^7^	6.5 (4.0–12.5)^14^	120.6 (4.3–920.0)^8^	10.8 (6.0–42.5)^9^	
UPCR (mg/g)	T1	73.9 (62.5–154.6)	126.1 (95.8–175.9)	311.3 (219.5–505.3)^12^	176.0 (137.0–181.1)^9^	^#^Group (*P* < 0.0001): FFC-DIA > FFC, SD, FFC-DIA + S
	T2	117.2 (76.2–132.2)^7^	96.1 (70.4–115.9)^14^	281.0 (130.2–461.1)^8^	115.9 (106.1–224.7)^9^	
UNGALCR (ng/mg)	T1	6.6 (4.0–9.3)^6^	13.9 (10.3–20.0)^14^	51.3 (37.1–122.6)^8^	27.0 (19.0–42.9)^8^	^#^Group (*P* < 0.0001): FFC-DIA, FFC-DIA + S > SD, FFC
	T2	5.0 (3.8–11.3)^6^	13.8 (8.5–19.5)^13^	38.9 (23.2–63.2)^8^	32.0 (11.6–53.7)^8^	

NGAL:neutrophil gelatinase-associated lipocalin, UACR: urinary albumin to creatinine ratio, UPCR: urinary protein to creatinine ratio, UNGALCR: urinary NGAL to creatinine ratio. Two parameters were influenced by cohort: Plasma creatinine (CoB > CoC) (P = 0.034) and plasma urea (CoA > CoC (P = 0.0003).

T1: Approx. 7 months of diet-feeding, T2: Approx. 13 months of diet feeding.

Median and interquartile range (Q1-Q3).

Superscript: Actual numbers included in the statistical analysis due to missing data. P-values from post hoc tests were adjusted using Bonferroni correction.

^#^Logaritmic transformation, ^##^Non parametric test.

### Haematology and clinical chemistry

FFC-DIA and FFC-DIA + S had lower sodium concentrations compared to FFC and SD. No significant differences were found in any of the other parameters (Supplementary information file 6).

### GFR by inulin clearance test

**Figure 2:**
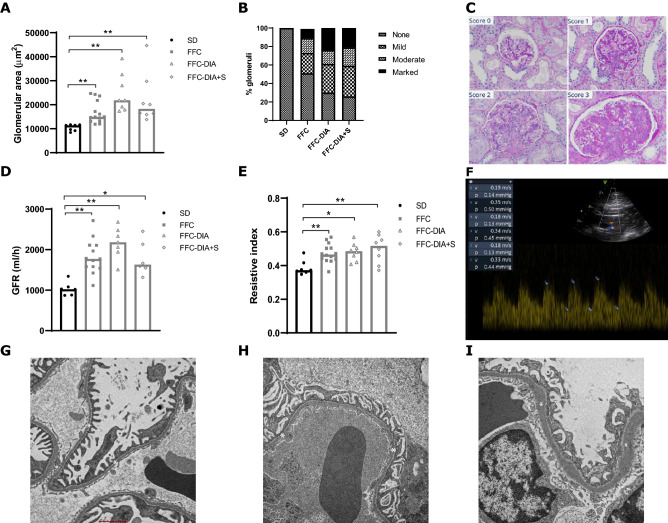
Data related to renal function and morphology. Glomerular size (**A**), mesangial expansion score (**B**), examples of mesangial expansion scores also illustrating the differences in glomeruli sizes (**C**). Score 0: no mesangial expansion, score 1: mild mesangial expansion, score 2: moderate mesangial expansion and score 3: marked mesangial expansion. Periodic acid-Schiff (PAS). × 40 original magnification. GFR estimated by inulin clearance test (**D**), resistive index (RI) (**E**), illustration of the RI measure (**F**), normal range glomerular basement membrane (*) in SD minipig, × 4800 original magnification (**G**) moderate thickening of basement membrane (*) in FFC minipig, × 4800 original magnification (**H**) severe thickening of basement membrane (*) and podocyte foot process fusion in FFC-DIA minipig, × 4800 original magnification (**I**). Castrated male Göttingen Minipigs fed with standard diet (SD) or fat, fructose and cholesterol rich diet (FFC) with or without additional salt (S) and with or without streptozotocin-induced diabetes (DIA). n = 6–14. *p < 0.05, **p < 0.01, ***p < 0.001.

The absolute GFR was significantly higher in FFC animals compared to SD and FFC-DIA at T1, and in all three FFC groups compared to SD at T2 (Fig. 2D). In addition, the GFR increased in all three FFC groups from T1 to T2 (Table 3, Supplementary information file 5).

**Table 3:** Kidney functional and morphological changes in castrated male Göttingen Minipigs fed with standard diet (SD) or fat, fructose and cholesterol rich diet (FFC) with or without additional salt (S) and with or without streptozotocin-induced diabetes (DIA).

Functional kidney parameters						
GFR (ml/min/pig)	T1	13.5 (12.8–14.9)	24.1 (19.6–26.2)^15^	17.9 (16.5–21.1)^10^	20.2 (14.7–23.5)^7^	Group*time (*P* < 0.0029): T1: FFC > SD, FFC-DIA T2:FFC, FFC-DIA, FFC-DIA + S > SD T2 > T1: FFC, FFC-DIA, FFC-DIA + S
	T2	17.1 (14.6–18.0)^8^	29.5 (26.3–33.1)^13^	36.4 (32.4–41.3)^7^	27.1 (25.8–35.6)^6^	
Resitive index	T1	0.42 (0.38–0.42)	0.44 (0.41–0.48)	0.46 (0.38–0.48)^12^	0.47 (0.43–0.55)^9^	**Group (*P* = 0.0002):** FFC, FFC-DIA, FFC-DIA + S > SD **Time (*P* = 0.047):** T2 > T1
	T2	0.37 (0.36–0.42)^7^	0.47 (0.44–0.50)^14^	0.49 (0.44–0.51)^8^	0.52 (0.43–0.57)^8^	

Kidney morphology						
Kidney weight (KW) (g)	T2	72 (57–76)^7^	116 (100–126)^14^	139 (128–151)^8^	109 (96–143)^8^	**Group (*P* < 0.0001):** FFC, FFC-DIA, FFC-DIA + S > SD
Kidney fibrosis	T2	0.0679 (0.0661–0.0104)^7^	0.0854 (0.0470–0.1259)^14^	0.0555 (0.0422–0.0722)^8^	0.0653 (0.0406–0.1152)^8^	**NS**
Average percentage glomeruli with ME score 0/1/2/3	T2	100/0/0/0	51/22/16/10^14^	30/31/15/24^8^	26/33/20/21	^**####**^**NS** for comparison between the FFC groups SD not included in the analysis since all had score 0
Glomerulus area (µm^2^)	T2	11,383 (9202–11,602)^7^	14,907 (13,174–21,771)^14^	21,839 (18,518–30,269)^8^	18,262 (16,066–25,095)^8^	^**##**^**Group (**P** < 0.0001):** FFC, FFC-DIA, FFC-DIA + S > SD

GFR: glomerular filtration rate, FFBM: fat-free body mass, KW: kidney weight. T1: Approx. 7 months of diet-feeding, T2: Approx. 13 months of diet feeding. Median and interquartile range (Q1-Q3). Superscript: Actual numbers included in the statistical analysis due to missing data. P-values from post hoc tests were adjusted using Bonferroni correction. ^##^Non parametric test, ^###^Two-sided analysis of variance, ^####^Proc genmod.

### 24 hour systemic blood pressure and heart rate

Thirty-nine animals were included in the mid study BP evaluations and 29 animals were included in the end study evaluations (see number of animals included in each group in Table 1). For all of DIA-BP, SYS-BP and MEAN-BP there were no significant differences between the groups; all values were higher at T2 compared to T1, and in the day-time compared to the night-time (Table 1, Supplementary information file 7). For HR, the values were also higher in the day-time versus the night-time, but lower at T2 compared to T1. In addition, the two FFC-DIA groups had significantly higher HR compared to both FFC and SD.

### Resistive index (RI)

There was a significant difference in RI between groups both at T1 and T2, with all three FFC groups having higher values than SD (Table 3, Fig. 2E). In addition, there was a significant overall increase in RI from T1 to T2.

### Kidney weight and histopathological evaluations of kidney tissue

The three FFC-groups had significantly higher absolute KW compared to SD (Table 3).

Based on Periodic acid–Schiff (PAS) staining, the kidneys in the three FFC groups showed varying degrees of histopathological changes such as hypertrophic and hyperplastic glomerular lesions and basement membrane thickening. Occasionally, glomerulosclerosis, periglomerular fibrosis, tubular atrophy and tubular basal membrane thickening associated to focal interstitial fibrosis were observed (n = 2 FFC; n = 2 FFC-DIA, Supplementary information file 8), but quantification of fibrosis using image analysis revealed no significant difference between the groups. Specifically, the glomerular size was significantly higher in all three FFC groups compared to SD (Fig. 2) and mesangial expansion evaluated as mesangial score was higher in all 3 FFC groups compared to SD (Fig. 2B). The lesions were not followed by clear immune cell infiltration or other characteristic features observed in diabetic nephropathy.

### TEM

The glomerular basement membrane thickening, observed by PAS staining in the histopathological evaluation, was confirmed by TEM (Fig. 2). Basement membrane thickening was observed both in the FFC and FFC-DIA groups but was most pronounced in the FFC-DIA group alongside with podocyte foot process fusion and nodular masses of mesangial matrix.

### Correlations

**Figure 3:**
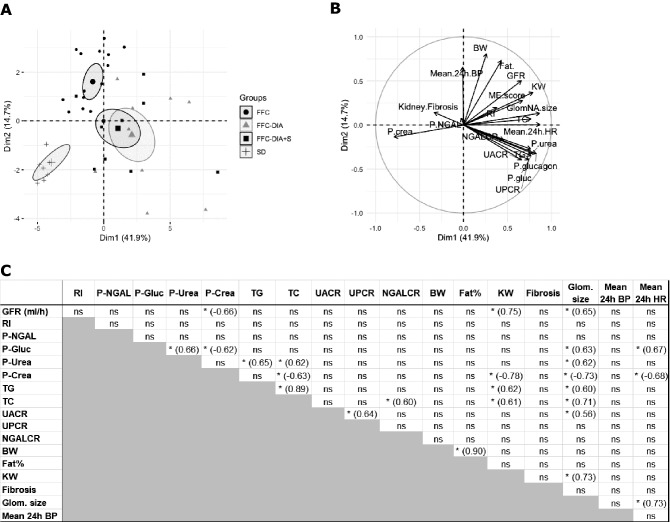
PCA plot (**A**), PCA variable correlation plot (**B**), and Spearman correlation analysis (**C**). *p < 0.05, number in parenthesis is the Spearman correlation coefficient for significant correlations. RI: resistive index, p-NGAL: plasma neutrophil gelatinase-associated lipocalin, p-Glu: plasma glucose, P-crea: plasma creatinine, TG: triglycerides, TC: total cholesterol, GFR: glomerular filtration rate, UACR: urinary albumin to creatinine ratio, UPCR: urinary protein:creatinine ratio, NGALCR: urinary neutrophil gelatinase-associated lipocalin to creatinine ratio, Fat%: body fat percentage, KW: Kidney weight, Glom. Size: glomerular size, BP: blood pressure, HR: heart rate.

In Spearman correlation analysis of selected parameters including plasma and urinary biomarkers, renal histology, KW, RI, BW, Fat%, mean 24 h BP and mean 24 h HR, only a few correlations remained significant after Bonferroni correction (Fig. 3C and Supplementary information file 9).

### Gene expression

**Figure 4:**
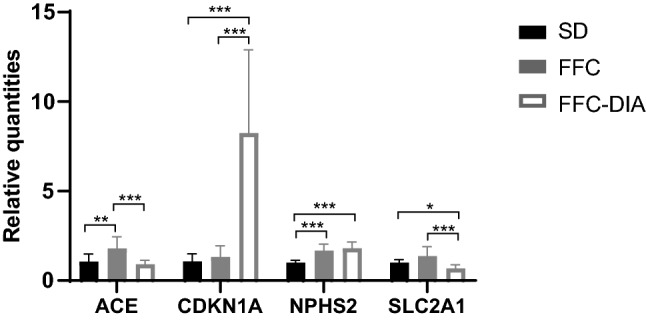
Gene expression data. Relative quantities of the 4 significantly deregulated genes (passing Bonferroni correction) in male, castrated Göttingen Minipigs fed with standard diet (SD, N = 7) or fat, fructose and cholesterol rich diet without induction of diabetes (FFC, N = 13) or with induction of diabetes (FFC-DIA, N = 8). Data are shown as mean ± SD, *p < 0.05, **p < 0.01, ***p < 0.001. Angiotensin I converting enzyme (*ACE*), cyclin dependent kinase inhibitor 1A (*CDKN1A*), solute carrier family 2 member 1 (*SLC2A1*) and podocin (*NPHS2*).

After qPCR data processing, 73 kidney related genes and 4 reference genes were expressed within the dynamic range in kidney tissue from the three diet groups SD, FFC and FFC-DIA and were accepted for further statistical analysis (see Supplementary information file 10 for raw data and data log2 FC for these 73 assays). Statistical analysis resulted in 4 genes being significantly de-regulated between groups after multiple test correction: Angiotensin I converting enzyme (*ACE*), cyclin dependent kinase inhibitor 1A (*CDKN1A*), solute carrier family 2 member 1 (*SLC2A1*) and podocin (*NPHS2*) (Fig. 4 and Supplementary information file 10).

### Principal components analysis (PCA)

The PCA plot revealed three major groups: diabetic minipigs, the FFC group and the SD group, respectively (Fig. 3A,B).

## Discussion

The present study investigated the effects of an FFC diet with and without chemically induced diabetes on renal functional and morphological features in a Göttingen Minipig model. Minipigs fed a FFC diet displayed some of the characteristic features of human ORG, such as glomerulomegaly, early hyperfiltration, moderate mesangial expansion and moderate thickening of the glomerular basement membrane, whereas induction of diabetes on top of the FFC diet, lead to further changes resembling the early stages of human DN, e.g. exacerbation of the glomerulomegaly and the glomerular basement membrane thickening, increased mesangial expansion, increased GFR, proteinuria and fusion of podocyte foot processes.

As expected, the FFC diet induced obesity in all diet-fed groups, as signified by higher BW and fat percentage. In addition, dyslipidemia with increased TG and TC was observed in all diet-fed groups as previously described in Ossabaw pigs fed a similar diet^21^. The presence of diabetes further exacerbated the TG as is also the case in humans^22^ and other pig models^23^. Hyperglycemia and increased fructosamine were only evident in the two diabetic groups, in line with many other reports from pigs showing no or only mild diet-induced increases in plasma glucose^24^. In humans, hypertension is often present in obese and diabetic individuals^25^ and is partly related to the dietary salt intake^26,27^. In the present study, feeding an FFC diet with or without concomitant presence of diabetes and with or without additional dietary salt did not lead to increased BP, which is in contrast to what has previously been observed in pigs fed a high fat, high sugar and/or salt-containing diet, indicating that the salt content and/or feeding period used here may have been insufficient to induce chronic hypertension^28,29^. However, in the current study, the presence of diabetes led to increased HR independent of the salt content of the diet and independent of increases in BP. Resting HR is described to be positively correlated to BP in young people^30^, but in this case the increased HR in the diabetic groups could be a result of autonomic neuropathy as has been suggested in humans^31^, although this needs further investigation.

In humans, obesity without diabetes leads to ORG which includes some of the same renal changes as seen in DN, e.g. glomerulomegaly, hyperfiltration, focal thickening of glomerular and tubular basement membranes, moderate mesangial expansion and podocyte effacement^4,5,32^, likely reflecting the many common risk factors. This is not surprising given that some of the main contributors to renal disease like obesity, insulin resistance, dyslipidemia, hypertension and low-grade inflammation are shared between obese and especially type 2 diabetic individuals^1,5^. In the present minipig model, glomerulomegaly was observed in all three FFC groups independent of diabetes, and in addition, the mesangial expansion score was higher in all the FFC groups compared to the SD group and with a tendency for more severe changes in the two diabetic groups. Mesangial expansion has been observed in other both obese and diabetic pig models^16,18^, which is in line with observations in humans, where moderate ME is observed in ORG^32^ and more severe ME is observed in DN^33^. Cyclin dependent kinase inhibitor 1A gene (*CDKN1A*) was found to be significantly upregulated in the diabetic animals compared to FFC and SD. CDKN1A regulates the cell cycle progression at the G_1_ and S-phase, and by inhibiting cyclin A, D and E it is able to arrest the cell in the G_1_ phase, leading to cell hypertrophy^34^. Specifically in DN, increased expression of *CDKN1A* has been related to glomerular/mesangial cell hypertrophy^35^ and podocyte apoptosis^36^, which aligns well with the glomerulomegaly and increased mesangial matrix observed primarily in the diabetic groups. None of the genes related to fibrosis or inflammation were significantly differentially regulated in line with the overall absence of fibrosis and inflammation in the renal sections.

In a few animals, more advanced diabetoid changes such as glomerulosclerosis, periglomerular fibrosis, tubular atrophy and tubular basal membrane thickening associated to focal interstitial fibrosis was observed, but the sporadic nature of these findings limits the value from a model perspective.

Glomerular filtration rate (Table 3), was increased in all FFC groups compared to SDat T2. Furthermore, this increase in GFR was correlated to the increased KW and glomerular size as judged by the positive correlations between KW, glomerular size and GFR (Fig. 3). The increased rather than decreased GFR has been observed in other obese and hyperglycemic pig models^18,37^ and in humans with obesity^38^ and early DN^39^, thus suggesting that the pigs in the present study are in the early stages of the disease.

The RI was increased to a similar extent in all the FFC groups, indicating a main effect of the diet rather than the diabetes. The increased RI may be related to the renal morphological and functional changes observed in all the FFC groups, but could also be related to systemic haemodynamic factors^40^, like e.g. the concurrent presence of atherosclerosis in the model^41^.

Like in other obese pig models, plasma creatinine was decreased in all FFC groups compared to SD^24^ which may be related to hyperfiltration, hypersecretion, decreased tubular reabsorption or differences in creatinine production from muscle creatine and/or differences in dietary creatine intake^7^. Since the FFBM was largely similar in all groups, reduced creatinine production from the muscles is an unlikely explanation, and the negative correlation between KW/glomerular size/GFR and plasma creatinine rather supports hyperfiltration as a cause of the lower plasma creatinine levels. This is also in line with the hyperfiltration observed both in ORG and in early stages of DN in humans^38,39^. Urea was increased in the two diabetic groups, which could indicate a significantly reduced renal function, although other factors like dietary protein intake together with net protein utilisation may also affect urea concentrations^42^. In dysregulated diabetic conditions, whole-body protein breakdown is increased^43^, and in light of the normal to increased GFR in the two diabetic groups, changes in protein metabolism may be a more plausible cause of the increased urea concentrations than severe renal dysfunction. This is supported by the higher plasma glucagon concentrations in the diabetic groups that correlated significantly with plasma urea concentrations (p < 0.01 at both time points, data not shown), an association that has also been reported in patients with type 1 diabetes^44^.

The plasma NGAL did not change in the current study, which may be due to the limited tubulointerstitial changes observed or to the timing of the blood samples since the plasma NGAL values tend to be primarily increased in the acute stages of renal disease^9^. Urinary NGALCR, on the other hand, was increased in the two diabetic groups at both time points compared to both FFC and SD, which indicate increased glomerular filtration and/or decreased tubular reabsorption of NGAL^9,45^. UPCR was only significantly increased in the FFC-DIA group compared to SD at T2 whereas UACR was not significantly different between any of the groups. This difference between levels of UNGALCR and UPCR/UACR may be explained by the different pathogenic processes causing their increase or simply by the high variability in especially the urinary albumin values. Some changes in the podocyte morphology, e.g. fusion of podocyte foot processes, were observed on EM in the FFC-DIA animals but since the podocyte changes were not quantified no correlations to the degree of albuminuria/proteinuria could be performed, which is a limitation of this study. In diabetic humans, podocyte dysfunction is associated with decreased expression of the two podocyte proteins, nephrin and podocin that are both involved in maintaining the glomerular filtration barrier^46^. In contrast, in this study, the *NPHS2* gene encoding podocin was significantly upregulated in both FFC and FFC-DIA vs. SD and the *NPHS1* gene encoding nephrin tended to be upregulated in the FFC vs. the SD but both with moderate fold changes. This could be due to the different stages of renal disease in the minipigs vs. the examined humans, since the humans were all in the later stages of the disease judged by the more consistent presence of proteinuria and the significantly decreased GFR^46^.

ACE and ACE2 are both involved in regulation of the renin-angiotensin system, with ACE2 being protective and ACE having deleterious effects with respect to development and progression of DN^47^. In the minipigs, *ACE* and *ACE2* expression was significantly higher in the FFC group compared to both SD and FFC-DIA whereas in human DN patients, *ACE* expression was found to be increased and ACE2 expression to be decreased both in the glomerular and the proximal tubular compartments^48^. Overall, it may be the balance between the expression of these two genes and their downstream effectors that are more important than the expression of the individual genes per se. The reduction in SLC2A1 in the diabetic minipigs is most likely a direct consequence of the increased plasma glucose levels^49^.

As described above, some parameters seemed to be influenced mainly by the diet and others mainly by the presence of diabetes, whereas in other cases the presence of diabetes seemed to exacerbate the effects of the diet. Despite this overlap between diet- and diabetes induced renal changes, the PCA plots with three distinct groups support a differentiation between the dietary effects and the diabetes effects. Extra dietary salt did not increase BP or exacerbate the renal changes, and thus no clear differentiation between the two diabetic groups was observed in the PCA plot. The lack of salt-induced hypertension is a limitation of the study, since part of the aim was to investigate if salt-induced hypertension exacerbated the renal changes. In addition, the inclusion of a lean hyperglycemic group would have completed the study, but since literature data on renal changes in purely hyperglycemic pigs indicate that only very early changes develop within a reasonable time frame, it was prioritised to combine the hyperglycemia with some of the other risk factors for developing diabetic nephropathy, such as diet-induced obesity/metabolic syndrome and hypertension, in order to accelerate the disease development.

Fifteen animals, primarily in the FFC-DIA groups, died spontaneously or were prematurely euthanised during the study which challenges the use of this model in long-term pharmacological intervention studies. However, the pigs in the present studies were used for other purposes and a more simple study design may prevent the issues.

## Conclusions

Göttingen Minipigs fed an FFC diet displayed some of the characteristic features of human ORG, such as glomerulomegaly, moderate ME and hyperfiltration and moderate thickening of the glomerular basement membrane. Induction of diabetes on top of the FFC diet, lead to further changes resembling the early stages of human DN, e.g. exacerbation of the glomerulomegaly, the ME and the glomerular basement membrane thickening, increased GFR, proteinuria and fusion of podocyte foot processes. In addition, UNGALCR was shown to be a relevant early biomarker of especially diabetes-related renal disease. The diet-fed Göttingen Minipig thus seems to be a relevant model for the early changes seen in both ORG and DN. Further studies are needed to show whether inclusion of e.g. hypertension in the model will induce some of the later disease stages.

## Supplementary Information

Supplementary Information 1.

Supplementary Information 2.

Supplementary Information 3.

Supplementary Information 4.

Supplementary Information 5.

Supplementary Information 6.

Supplementary Information 7.

Supplementary Information 8.

Supplementary Information 9.

Supplementary Information 10.

## Data Availability

The datasets used and analysed during the current study are available from the corresponding author on reasonable request. Gene expression datasets generated and analysed during the current study are available in the Gene Expression Omnibus (GEO) repository, accession number GSE215843.

## Abbreviations

BP: Blood pressure
BW: Body weight
DEXA: Dual energy x-ray absorptiometry
DIA-BP: Diastolic blood pressure
DN: Diabetic nephropathy
Fat%: Total body fat percentage
FFBM: Fat-free body mass
FFC: Fat, fructose and cholesterol diet
FFC-DIA: Minipigs fed FFC diet and chemically induced with diabetes
FFC-DIA + S: Minipigs fed FFC diet with 2.5% salt and chemically induced with diabetes
GFR: Glomerular filtration rate
HR: Heart rate
KW: Kidney weight
LLOQ: Lower limit of quantification
LOCI: Luminescent oxygen channeling assay
ME: Mesangial expansion
MEAN-BP: Mean blood pressure
NGAL: Neutrophil gelatinase-associated lipocalin
NGALCR: Urinary NGAL to creatinine ratio
ORG: Obesity-related glomerulopathy
PAS: Periodic Acid-Schiff
PCA: Principal components analysis
PSR: Picro-Sirius Red
RI: Resistive index
ROI: Region of interest
STZ: Streptozotocin
SYS-BP: Systolic blood pressure
TEM: Transmission electron microscopy
TC: Total cholesterol
TG: Triglyceride
UACR: Urinary albumin to creatinine ratio
UPCR: Urinary protein to creatinine ratio
