# Supplementary material for: Functional and morphological renal changes in a Göttingen Minipig model of obesity-related and diabetic nephropathy
Source: Sci Rep. 2023 Apr 12;13:6017. doi: 10.1038/s41598-023-32674-6 (PMC10097698; doi:10.1038/s41598-023-32674-6)
Supplement: Supplementary file 1 — Supplementary Information 1. [file 41598_2023_32674_MOESM1_ESM.docx]

**Supplementary information file 1: Publications based on same animals**

Following publications are based on the same animals. These publications include parts of the baseline characteristics of the SD and FFC groups.

L.J. Andreasen, S. Krog, T.P. Ludvigsen, O.L. Nielsen, J.E. Møller, B. Christoffersen, H.D. Pedersen, L.H. Olsen, Dietary normalization from a fat, fructose and cholesterol-rich diet to chow limits the amount of myocardial collagen in a Göttingen Minipig model of obesity, Nutrition & metabolism 15 (2018) 64.

T.P. Ludvigsen, S.F. Pedersen, A. Vegge, R.S. Ripa, H.H. Johannesen, A.E. Hansen, J. Löfgren, C. Schumacher-Petersen, R.K. Kirk, H.D. Pedersen, B. Christoffersen, M. Ørbæk, J.L. Forman, T.L. Klausen, L.H. Olsen, A. Kjaer, (18)F-FDG PET/MR-imaging in a Göttingen Minipig model of atherosclerosis: Correlations with histology and quantitative gene expression, Atherosclerosis 285 (2019) 55-63.

C. Schumacher-Petersen, B. Christoffersen, R.K. Kirk, T.P. Ludvigsen, N.E. Zois, H.D. Pedersen, M. Vyberg, L.H. Olsen, Experimental non-alcoholic steatohepatitis in Göttingen Minipigs: consequences of high fat-fructose-cholesterol diet and diabetes, Journal of translational medicine 17(1) (2019) 110.

T.P. Ludvigsen, L.H. Olsen, H.D. Pedersen, B. Christoffersen, L.J. Jensen, Hyperglycemia-induced transcriptional regulation of ROCK1 and TGM2 expression is involved in small artery remodeling in obese diabetic Göttingen Minipigs, Clinical science (London, England : 1979) 133(24) (2019) 2499-2516.

S. Cirera, E. Taşöz, M. Juul Jacobsen, C. Schumacher-Petersen, B. Østergaard Christoffersen, R. Kaae Kirk, T. Pagh Ludvigsen, H. Hvid, H. Duelund Pedersen, L. Høier Olsen, M. Fredholm, The expression signatures in liver and adipose tissue from obese Göttingen Minipigs reveal a predisposition for healthy fat accumulation, Nutrition & diabetes 10(1) (2020) 9.

Y. Feng, S. Cirera, E. Taşöz, Y. Liu, L.H. Olsen, B. Christoffersen, H.D. Pedersen, T.P. Ludvigsen, R.K. Kirk, C. Schumacher-Petersen, Y. Deng, M. Fredholm, F. Gao, Diet-Dependent Changes of the DNA Methylome Using a Göttingen Minipig Model for Obesity, Frontiers in genetics 12 (2021) 632859.

L.B. Christiansen, T.L. Dohlmann, T.P. Ludvigsen, E. Parfieniuk, M. Ciborowski, L. Szczerbinski, A. Kretowski, C. Desler, L. Tiano, P. Orlando, T. Martinussen, L.H. Olsen, S. Larsen, Atorvastatin impairs liver mitochondrial function in obese Göttingen Minipigs but heart and skeletal muscle are not affected, Scientific reports 11(1) (2021) 2167.

In addition, the following posters have been presented at scientific congresses:

S Krog, OL Nielsen, JE Møller, TP Ludvigsen, HD Pedersen, UT Baandrup, LH Olsen; Myocardial fibrosis and fat infiltration in obese and diabetic Göttingen Minipigs. Poster presentation at the international meeting Heart Failure, Paris, 2017.

LJ Andreasen LJ, S Krog, TP Ludvigsen, JE Møller, HD Pedersen, OL Nielsen, LH Olsen, Cessation of high-fat diet lowers the degree of myocardial fibrosis in obese Göttingen Minipigs. Poster presentation at the international meeting Metabopig - Metabolic Research in Minipigs, Lille, France 2017.

C Schumacher-Petersen BØ Christoffersen, RK Kirk, TP Ludvigsen, NE Zois, HD Pedersen, M Vyberg, LH Olsen. Characterization of hepatic changes in a diet-induced obese minipig model. Poster presentation at International conference on Fatty Liver, Seville, Spain, 2017.
