# Supplementary material for: Functional and morphological renal changes in a Göttingen Minipig model of obesity-related and diabetic nephropathy
Source: Sci Rep. 2023 Apr 12;13:6017. doi: 10.1038/s41598-023-32674-6 (PMC10097698; doi:10.1038/s41598-023-32674-6)
Supplement: Supplementary file 2 — Supplementary Information 2. [file 41598_2023_32674_MOESM2_ESM.docx]

**Supplementary information file 2: Nutritional composition of the experimental diets**

|  | Standard diet^a^ | FFC 2% cholesterol (5B4L)^b^ | FFC 1 % cholesterol (9G4U)^b^ | FFC 1 % cholesterol and 2.5 % added NaCl  (5BTJ)^b^ |
| --- | --- | --- | --- | --- |
| Carbohydrates (%)  Protein (%)  Fat (%)  Fructose (%)  Cholesterol (ppm)  Methionine (ppm)  Choline (ppm)  NaCl equivalents  Cholate (% w/w)  Energy (kcal/g) | 74.6  18.6  6.8  5.5 (all sugars)  ~ 0  1800  784  0.73  No added  2.8 | 40.8  16.2  43  17.8  20,045  3500  668  0.65  0.7  4.14 | 40.8  16.1  43  18.8  10,045  3500  668  0.65  0.7  4.14 | 40.3  16.1  43.6  18.8  10,045  3500  639  2.5  0.7  4.07 |

Percentages are of total energy content unless otherwise noted, ppm: parts per million, NaCl equivalents: calculated as 2.5 times the sodium content

^a^ Minipig, Special Diet Services (SDS), Essex, UK

^b^ TestDiets®, Missouri, USA
