# Supplementary material for: Functional and morphological renal changes in a Göttingen Minipig model of obesity-related and diabetic nephropathy
Source: Sci Rep. 2023 Apr 12;13:6017. doi: 10.1038/s41598-023-32674-6 (PMC10097698; doi:10.1038/s41598-023-32674-6)
Supplement: Supplementary file 3 — Supplementary Information 3. [file 41598_2023_32674_MOESM3_ESM.docx]

**Additional file 3: More detailed description of the methods used in the study.**

**SURGICAL METHODS AND ANAESTHESIA**

| **Procedure** | **Description** |
| --- | --- |
| Anaesthesia | Implantation of central-venous catheters/blood pressure transmitters was done in 18 h fasted animals under general anaesthesia induced by 1 ml/10-15 kg of a mixture containing 125 mg zolazepam and 125 mg tiletamin (Zoletil® 50 Vet, ChemVet, Denmark), ketamine (1.25 mL Ketaminol® Vet 100mg/mL, MSD Animal Health, Denmark), xylazin (6.5 mL Rompun Vet, 20 mg/mL, Bayer A/S, Denmark) and butorphanol (2.5 mL Torbugesic®, 10 mg/mL, Scanvet, Denmark)). This could be supplemented with 1/3 of the initial dose if needed for the longer procedures. For surgical implantation of catheters and blood pressure transmitters the anaesthesia was maintained on inhalation anaesthesia (oxygen and isoflurane 1-2%). |
| Antibiosis and analgesia | For ultrasound-guided and surgically implanted venous catheters and blood pressure transmitters, Noropen prolongatum was given pre-operatively IM in a dose of 1 ml/ 15 kg for peri-and post-operative antibiosis. Post-surgical analgesia was obtained by buprenorphine (Vetergesic® Vet. 0.3 mg/ml, Orion Pharma Animal Health) given IM in a dose of 0.05-0.17 mL/kg just after completion of the surgical procedure and Metacam® (20 mg/ml, Boehringer Ingelheim Animal Health Nordics A/S) given IM once daily for 3 days post-surgery.  For ear vein catheters Metacam® (20 mg/ml, Boehringer Ingelheim Animal Health Nordics A/S) was given IM once daily for 3 days for post-implantation analgesia. |
| Semi-permanent centralvenous catheter implanted through the ear vein | A central venous catheter (BD Careflow 3Fr 200mm, Argon Medical, Texas, USA) was implanted in v. jugularis via an ear vein using Seldingers technique. The tip of the catheter ended in v. jugularis close to the cranial caval vein. The external part of the catheter was fixed to the pinna using medical tape or with a plastic plate and steel piercings.  Pentrexyl (1g/10 ml sterile saline, 1ml/10kg) was given IV in the catheter before closure as described below.  In some cases, e.g. after diabetes induction, the catheter was removed again allowing group housing of the animals and minimising the need for catheter maintenance and the risk for catheter-related infections. |

| Surgical implantation of central-venous catheter | A single lumen catheter (C-TPNS-6.5-90-REDO, William Cook Europe Aps; Bjaeverskov, Denmark) was implanted surgically. The catheter was tunneled subcutaneously from the neck and down to the jugular groove, where an incision was made in the skin. The jugular vein was dissected and retracted up into the incision. A small incision was made in the vein, the catheter was introduced and advanced 10 cm into the vein with the tip ending in or close to the cranial caval vein. A fixing suture was applied around the vein securing the catheter in this position. The catheter was flushed with 10 ml sterile saline and closed as described below. The surgical incision was closed in 3 layers, and a bandage was applied to protect the external part of the catheter. |
| --- | --- |
| Ultrasound-guided implantation of  central venous catheter | A linear array transducer (13 MHz) was placed in the jugular groove with the pig in dosal recumbency. A 5 ml syringe attached to a hypodermic valve needle was guided into the vein. A guide wire was introduced through the valve needle’s second Luer port. As the guide wire was advanced into the vein, the valve needle and attached syringe was retracted and removed. A dilator was used to facilitate percutaneous penetration, whereafter an antimicrobially coated double-lumen catheter (Certofix Protect Duo V730, BBraun) was advanced over the guide wire, until the desired length was inserted (catheter-tip ending in the cranial caval vein). A fixation wing was used to secure the catheter to the skin by means of polypropylene sutures. |
| Closure of catheters after flushing/blood sampling | The catheter was flushed with 10 ml of sterile 0.9 % NaCl. On days with multiple blood samples, additionally 0.1 mL/kg Pentrexyl inj. (ampicillin, 1 g dissolved in 10 mL sterile saline) was injected IV in the catheter. Finally, the catheter was filled with 0.8-1.2 ml (depending on the catheter type) TauroLock Hep500 (Nordiatech A/S, DK) and closed with a new luer-lock. |

| Blood pressure transmitters | An incision was made in the jugular groove, and the carotid artery was isolated. Two holding ligatures were placed loosely around a. carotis and the artery was drawn up in the incision. A pursestring suture was prepared (Ethibond Excel 4-0) and a small incision was made in the artery with needle (size 16G). The telemetry sensor was introduced in the artery and advanced 9-10 cm to an expected position with the tip placed just inside the aortic arch. The pursestring suture was gently tied ensuring haemostasis, and some drops of glue (3M Vetbond) were placed around the sensor. In some cases an approx. 1 x 1 cm Cellulose patch was placed around the site of insertion of the sensor into the artery to prevent seeping of blood, and in other cases a ligature was placed around the sensor and the artery for haemostasis and securing the sensor position. The incision was closed in 3 layers, and a pressure bandage was applied for 3-5 days.  Following blood pressure measurements, the transmitters were surgically removed during general anaesthesia as described above. In most cases, haemostasis could be obtained by compressing the artery for 20 minutes, but otherwise the incision in the artery was closed by suture. The surgical wound was closed in 3 layers. |
| --- | --- |

**IN VIVO EVALUATIONS AND SAMPLING OF BLOOD AND TISSUES**

| **Measurement** | **Method** |
| --- | --- |
| Body weight and DEXA scanning | Body weight (BW) was obtained by weighing the animals once weekly throughout the study and in connection with the various tests. Total body fat percentage (Fat%) and fat-free body mass (FFBM) were estimated at T1 and T2 using dual energy x-ray absorptiometry (DEXA) scanning (Lunar prodigy, GE Healthcare, Brøndby, Denmark). The scanning was performed in 18 h fasted animals under general anaesthesia induced by 1 ml/10-15 kg of a mixture containing 125 mg zolazepam and 125 mg tiletamin (Zoletil® 50 Vet, ChemVet, Denmark), ketamine (1.25 mL Ketaminol® Vet 100mg/mL, MSD Animal Health, Denmark), xylazin (6.5 mL Rompun Vet, 20 mg/mL, Bayer A/S, Denmark) and butorphanol (2.5 mL Torbugesic®, 10 mg/mL, Scanvet, Denmark)). This could be supplemented with 1/3 of the initial dose if needed for the longer procedures |
| Blood sampling | Blood sampling for measurement of selected metabolic parameters and renal biomarkers was performed in animals fasted for approx. 18 h. Blood was obtained through the permanent IV catheters and transferred into plain serum tubes or EDTA coated tubes. Samples were kept at room temperature for 1 hour (serum samples) or placed in wet ice for a maximum of 30 minutes (EDTA coated tubes) prior to centrifugation (2000G, 10 mins, 4°C). The resulting plasma and serum were stored at -20°C (insulin, glucagon and C-peptide) or -80°C (remaining parameters) until analysis as described below. In addition, at study termination, blood samples were drawn from the jugular vein and transferred into EDTA coated and plain serum tubes. The samples were immediately analysed at The Veterinary Diagnostic Laboratory, Department of Veterinary Clinical Sciences, University of Copenhagen, where a complete blood count was obtained from EDTA stabilised blood, while biochemical analyses were performed on serum. |
| Inulin clearance test | The animals were fasted for approx. 18 hours prior to the inulin clearance and last injection of insulin was given 48 h prior to the test. Inulin (Inulin-FITC, TdB Consultancy AB, Sweden) was given as an IV bolus injection (dose 5 mg/kg at T1 and 2.5 mg/kg at T2) and followed by flushing with sterile saline. Blood was sampled into EDTA coated tubes and kept on wet ice for a maximum of 30 minutes before centrifugation (2000G, 10 mins, 4°C). The resulting plasma was kept at -80 ºC until analysis for inulin content as described below. The inulin clearance was calculated by non-compartmental pharmacokinetic analysis (Phoenix WinNonlin, Pharsight, Mountain View, CA, USA). |
| Blood pressure measurement by telemetry | DSI™ PA-C10 Blood Pressure Telemetry Transmitter and telemetry equipment was used for the systemic BP measurements. Ponemah 5.20-SP1 software was used for BP recordings and analysis (telemetry equipment and software both Data Sciences International (DSI, St. Paul, MN, USA). BP transmitters were implanted in the right carotid artery with the tip ending in the aortic arch as described in Supplementary Table S2. Systolic BP, diastolic BP and mean BP as well as heart rate (HR) was recorded every 10 seconds for 24 hours at T1 and T2 (Figure 1B). The animals were left as undisturbed as possible for the 24 hours of continuous BP measurements. Thirty-minutes averages of SYS-BP, DIA-BP, MEAN-BP and HR were calculated. Recording periods with missing data and/or poor signal quality/noise were excluded. |
| Resistive index (RI) | RI ultrasound recordings and urine sampling were performed under general anaesthesia at T1 and T2. RI recordings were obtained using the Vivid I ultrasound scanner with a 5S-RS sector transducer (GE Healthcare, Brøndby, Denmark) with animals placed in lateral recumbency. Colour Doppler flow imaging of the central part of the kidney was used to localize a central interlobar artery and peak systolic and diastolic velocity were recorded using pulsed wave Doppler imaging. Continuous electrocardiography was included. Offline analyses were performed in EchoPac (EchoPac Software only, version 113, GE Healthcare, Brøndby, Denmark) with the operator blinded to pig group. RI was calculated from three consecutive pulse waveforms ((systolic peak velocity – end diastolic velocity) / systolic peak velocity * 100). An average of these was made for each kidney, and these were subsequently averaged to obtain a global RI for each animal. Inter- and intra-observer repeatability regarding global RI was assessed in 6 randomly selected animals, by two observers (each observer measured six animals six times). The mean inter-observer variability was 7.4 % (range: 2.9 - 12.2 %) and intra-observer variability was 2.1 % (range: 0.9 - 3.9 %). |
| Urinary sampling by cystocentesis | Urinary samples were obtained by ultrasound-guided cystocentesis in the same anaesthesia as the RI recordings. Samples were kept in wet ice for a maximum of 60 minutes prior to centrifugation (2000G, 10 mins, 4°C) and stored at -80°C until further analysis as described below. |
| Tissue sampling at termination | Both kidneys were harvested, weighed and de-capsulated. Two cortical tissue-samples for gene expression were obtained from the central part of the left kidney; one was snap-frozen in liquid nitrogen and thereafter kept at -80^o^C and the other was placed in RNA-later and kept at 5^o^C for approx. 24 h and thereafter at -20^o^C. Tissue samples containing both medulla and cortex were obtained from the central part of both kidneys, immersion fixed in 4% paraformaldehyde for 24-48 h, processed by standard procedures through graded concentrations of alcohol and xylene and lastly embedded in paraffin for later histopathological analysis as described below.  In addition, cortical and medullary tissue from the central part of the right kidney was sampled for transmission electron microscopy (TEM) and placed in cold Karnowsky´s fixative until further processing. |

**PLASMA ANALYSES**

| **Plasma analysis** | **Method** |
| --- | --- |
| Plasma glucose, TG TC, fructosamine, creatinine and urea.  Urine creatinine, protein and glucose | Plasma levels of glucose, fructosamine, triglycerides (TG), total cholesterol (TC), creatinine, urea and albumin together with urinary concentrations of creatinine, protein and glucose were analysed using a Cobas 6000® autoanalyzer according to the manufacturer´s instructions (Roche A/S, Hvidovre, Denmark). |
| PLasma and urine NGAL | NGAL in both plasma and urine was evaluated using Pig NGAL ELISA Kit 044 according to the manufacturer’s instructions (BioPorto Diagnostics A/S, Hellerup, Denmark). In the process of in-house validation, intra-assay coefficient of variation (CV) (plasma 2.1% and urine 6.4%) was assessed by 12 folded measurement of 2 porcine plasma and 1 porcine urine sample at the same assay. Inter-assay CV (plasma 10.4% and urine 7.0%) was assessed by a double folded measurement of 3 plasma and 3urine samples at five (plasma) or six (urine) different assays. |
| Plasma insulin | Insulin was measured using Luminescence Oxygen Channeling Immunoassay (LOCI/AlphaLisa) by applying a mixture of biotinylated mAb Oxi-005 and mAb HUI-018-conjugated acceptor beads, lower limit of quantification was 3 pM.  The assay was developed at Novo Nordisk A/S. |
| Plasma glucagon | Glucagon content were determined using LOCI/ AlphaLisa by applying a mixture of biotinylated mAb GLU 2F7 and mAb GLU 1F120-conjugated acceptor beads, lower limit of quantification was 4 pM.  The assay was developed at Novo Nordisk A/S. |
| Plasma inulin | Plasma inulin levels were analysed as follows: Calibrators with FITC-inulin were prepared in Göttingen Minipig plasma (Bioreclamation, MGP10358) at the following concentrations, 700000, 269231, 103550, 39827, 15318, 5892, 2266, 872, 335, 129, 50 and 0 ng/ml. All samples and calibrators were subsequently diluted 30-fold in 500 mM Hepes pH 7.4 containing 0.1 % Tween-20, and then 10 µl samples were added in duplicate to a black 384 well plate (In-Vitro, GR-788-076). Calibrators were added in quadruplicates. The plate was measured in an EnVision® 2102 Multilable Reader at the following instrument settings: excitation/emission 485/535 nm, measurement height 9 mm, no. of flashes 10, light intensity 20%, and Gain 155. Data was analysed with an internally developed application, LOCI Calculator, which offers a graphical user interface to the R environment for statistical computing (www.r-project.org). Standard curves were fitted using a 5-parameter logistic model with a 1/Y2 weighing function. The data exclusion criteria were determined by the lower limit of quantification (LLOQ) and the highest calibrator concentration 700000 ng/ml. The LLOQ was the highest value at which the calculated CV was less than 20% and the recovery of each calibrator was within 80-120% of the known value. Based on three separate LLOQ determinations in pig plasma this value was set conservatively to 1000 pg/ml, meaning that not all the calibrators were required for calculation.  The assay was developed at Novo Nordisk A/S. |
| Urinary albumin | Urinary albumin content was analysed using a commercial pig albumin ELISA kit according to the manufacturer´s instructions (Cat. No. E101-110, Bethyl Laboratories, [www.bethyl.com](http://www.bethyl.com)).  In the process of in-house validation, intra-assay coefficient of variation (CV) was assessed to 4.1% by 12-folded measurement of 4 different porcine urine samples at the same assay. Inter-assay CV was assessed to 2.9% by a double folded measurement the same 4 urine samples at 3 different assays. |

**HISTOPATHOLOGY AND ELECTRON MICROSCOPY**

| **Parameter** | **Method** |
| --- | --- |
| Histopathological examination | Paraffin sections of 3 microns were stained with Periodic Acid-Schiff (PAS) and scanned using the Nanozoomer 2.0 (Hamamatsu Photonics K.K., Hamamatsu, Japan) at a magnification of ×40.  The mesangial expansion (ME) was evaluated in a blinded fashion as 20 glomeruli of each kidney evenly distributed in the cortex were assessed and graded into four categories: 0 (no ME), 1 (mild ME, ME width < 2 nucleus diameter), 2 (moderate ME, ME width < 4 nucleus diameter), and 3 (severe ME, ME width > 4 nucleus diameter)(Figure 2C). Only glomeruli with a vascular and a urinary pole were assessed for ME.  Additionally, a general morphological assessment of changes in the kidneys was performed.  The extent of fibrosis in each kidney section was assessed on a Picro-Sirius Red (PSR) stained section. Specifically, all PSR slides were scanned using the Nanozoomer 2.0 at an original magnification of ×40. The image analysis was performed using Visiopharm Integrator System software (VIS; Visiopharm, Hoersholm, Denmark). An automated tissue detection protocol was performed. Evaluation of the collagen content (glomerular as well as extra-glomerular) in the PSR stain was determined in a region of interest (ROI) restricted to the cortex region. Within the ROI a threshold analysis was performed using the features “Contrast red-green median” (65-∞) and “Chromaticity green mean” (0-0.3). |
| Glomerular size | Mean glomerular size was measured by training of a deep-learning network. Specifically, the dataset included 37 digital images which randomly were divided into a training set consisting of 30 images (80 %) and a test set consisting of 7 images (20 %). The images were imported into the image analysis platform HALO (v3.1.1076.405, Indica Labs, Albuquerque, NM, USA). A new classifier was developed using “DenseNet AI” plugin, a deep learning classifier. The classifier included 2 classes including tissue, and glomeruli. The classifier was trained by labelling tissue structures into the different classes in the training data set. Approximately 10 glomeruli from each training image were labelled “glomeruli” and the tissue surrounding the glomeruli was labelled “tissue”. The performance of the classifier was evaluated by running the classifier on the test images. If some structures were not correctly identified, more labelling and training was performed based on the identified errors. This workflow was followed until the classifier identified the correct structures on all the test images. The classifier was used to calculate the area of all glomeruli of each tissue section (image) and the mean glomerular area was then calculated |
| TEM | Based on the histopathological evaluation 2 animals from each of the three diet groups SD, FCC, and FFC-DIA, were selected for TEM. The tissue from these animals was processed for TEM. In short, the tissue was trimmed into 1 mm^3^ pieces and fixed in cooled 5% glutaraldehyde and 4% formaldehyde in 0.064M sodium phosphate buffer, pH 7.2 overnight and post-fixed in 2% OsO_4_ in cacodylate buffer (Ampliqon, Denmark) for 1 hr at RT followed by a wash in cacodylate buffer and staining with 0.5% uranyle acetate (Sigma-Aldrich, Denmark) before processing to epon (Ax-Lab, Denmark) blocks using increasing gradients of ethanol and increasing concentrations of epon in propylene oxide (Sigma-Aldrich, Denmark). Ultra-thin sections (~70 nm), cut with a diamond knife (Diatome 3 mm) and collected at copper grids, mesh 200, were examined in a Tecnai G2 SpiritBT Transmission Electron Microscope (FEI, The Netherlands). Images were obtained with a CETA camera 16M 300kV, camera firmware version 6442, using Tecnai TEM Imaging & Analysis version 5.7.1 software. |

**GENE EXPRESSION**

| Gene expression analysis | For this part of the study the following animals and diet groups were included: SD (n=7), FFC (n=14) and FCC-DIA (n=8). Ninety-six genes chosen from an *in-house* panel (see Supplementary Table S4 for genes included in the study and the corresponding designed qPCR primers), including 4 reference genes (ACTB, TBP1 and RPL4), were profiled by high-throughput qPCR.  Sixty mg of kidney tissue per sample were homogenized in a GentleMACS^TM^ Octo Dissociator machine (Miltenyi Biotec) using the RNeasy® Mini Kit (Qiagen) with DNase digestion, following manufacturer’s instructions. Quantity and quality were evaluated by: 1) NanoDrop^TM^ 1000 Spectrophotometer (Thermo Fisher Scientific) measurement to obtain concentration and purity (260/280 and 260/230 ratios); 2) Experion^TM^ Automated Electrophoresis System (Bio-Rad) measurement to obtain a RNA quality Index; 3) visual inspection of RNA samples run in an agarose gel electrophoresis. Five-hundred ng of total RNA were used to perform cDNA synthesis following the protocol described in Mentzel et al. 2018 {Mentzel, 2018 #330}. Two cDNA replicates were synthesized for each RNA sample and diluted 16 times before used in quantitative real-time PCR (qPCR).  Assays were first optimized in a MxPro machine (Stratagene) and further optimized in a FlexSix IFC chip before running one 96.96 Dynamic Array IFC chip in a BioMark^TM^ HD platform (Fluidigm) following manufacturer’s protocol. Briefly, cDNA was pre-amplified using TaqMan PreAmp Master Mix (Applied Biosystems) and 16 cycles. Residual primers were digested using Exonuclease I (New England Biolabs). Subsequently, qPCR was carried out using 2X SsoFast EvaGreen Supermix with low ROX (Bio-Rad), primer pairs, 2X Assay Loading Reagent (Fluidigm) and 20X DNA Binding Dye (Fluidigm) according to manufacturer’s protocol. Thermal cycling conditions including melting curve analysis were 30 cycles of 5 seconds at 96ºC and 20 seconds at 60ºC. Finally, melting curve analysis was carried out by increasing the temperature from 60 to 95ºC to ensure specific amplification.  Raw qPCR data were manually curated by visual inspection of all melting curves. The cDNA replicates were accepted when the Cq values where < 1.5 cycles apart. When looking all assays, cDNA samples with > 20% of assays having the replicates >1.5 cycles apart were excluded. The PCR efficiencies were calculated based on the slope of a standard curve made with a dilution serial of a pool of all undiluted cDNA pre-amplified samples and only assays having a PCR efficiency between 80-115% were accepted for further processing. Subsequently, data were pre-processed using the GenEx Pro software version 6.1. Briefly, data were corrected for PCR efficiency (assays with an efficiency of 90-110% and R^2^≥ 0.98 were accepted), *ACBT*, *TBP* and *RPL4* were the most stable reference genes and were used to normalize the qPCR data. Subsequently, relative expression (or fold changes) for each assay was calculated scaling all samples to the SD diet group. Data were log2 transformed prior to statistical analysis. |
| --- | --- |
| Statistical analyses | For response variables evaluated both at T1 and T2 (BW, total body fat, systemic blood pressure (SYS-BP, DIA-BP, MEAN-BP), HR, circulating and urine biomarkers), repeated measurements models (PROC MIXED procedure) were used to account for correlations between measurements on the same animal. Group, cohort and time (T1 or T2) were included as class variables. Moreover, interaction between group and time was included as explanatory variable. An extra time class variable corresponding to the 30 minutes averages during the 24 hours continuous recording (n=48) was included for the HR and systemic blood pressure analyses, and day (12-17 PM) and night (00-05 AM) periods were defined. Pig was included as random variable. Two-way ANOVA with group and cohort as class variables was used to analyse data only recorded at study end (kidney weight, glomerular size, renal fibrosis and haematology). Logarithmic transformation was needed for some response variables to meet model requirements. The models were reduced with a backwards stepwise approach. Post hoc t-tests were performed if overall significance was obtained. Non-parametric Kruskal Wallis test was used if model requirements could not be obtained by logarithmic or square root transformation and post hoc Wilcoxon signed rank tests were used as non-parametric post hoc tests if overall significance was obtained. All post hoc *P*-values were Bonferroni adjusted for multiple testing. Mesangial matrix expansion recorded from 20 glomeruli per animal was analysed using the proportional odds model as the outcome variable was ordinal with 4 categories and the repeated measures structure was taken into account using a working independence assumption in PROC GENMOD. *P*-values<0.05 were considered significant.  Spearman correlation analyses were performed to test for associations between body/organ measures (BW, Fat% and kidney weight), renal *in vivo* function (RI index and GFR), plasma parameters (glucose, TG, TC, creatinine, urea and NGAL) and urine biomarkers (albumin, protein and NGAL, all adjusted to urine creatinine concentrations), histopathological changes (mean glomerular size and fibrosis), 24 hours telemetry measurements (MEAN-BP and HR). *P*-values were adjusted by Bonferroni correction.  For qPCR data, statistical analysis of the kidney-related genes (excluding reference genes) was performed using one-way ANOVA with group as class variable. Significance levels were set to p=0.0007 to account for performing multiple comparisons (corrected *P*-values, done by the GenEx Pro software). Moreover, only fold changes ≥1.5 (up or down) were considered biologically relevant and discussed further (Supplementary table S4).  To further visualize grouping within the datasets, a principal component analysis (PCA) including relevant phenotypical variables (BW, Fat%, KW, RI, plasma NGAL, plasma glucose, glucagon, urea, creatinine, total cholesterol and TG, UACR, UPCR, NGALCR, Fibrosis area, glomerular size, mesangial expansion score, mean 24h BP and mean 24 h HR) was conducted using the R package FactoMineR and the package missMDA to deal with missing values (Figure 4 A). The most contributing variables to the variation in dimension 1 and 2, respectively, were identified and shown on Figure 4B. |
