# Supplementary material for: Functional and morphological renal changes in a Göttingen Minipig model of obesity-related and diabetic nephropathy
Source: Sci Rep. 2023 Apr 12;13:6017. doi: 10.1038/s41598-023-32674-6 (PMC10097698; doi:10.1038/s41598-023-32674-6)
Supplement: Supplementary file 4 — Supplementary Information 4. [file 41598_2023_32674_MOESM4_ESM.docx]

**Additional file 4: Inulin curves used for calculation of glomerular filtration rate (GFR)**

Inulin vs. time profiles in male, castrated Göttingen Minipigs fed with standard diet (SD) or fat, fructose and cholesterol rich diet (FFC) with or without additional salt (S) and with or without streptozotocin-induced diabetes (DIA). T1 (A, B) and T2 (C, D). Left panel: ordinary scale, right panel: log scale. Mean±SEM, n=6-15.
