# Supplementary material for: Functional and morphological renal changes in a Göttingen Minipig model of obesity-related and diabetic nephropathy
Source: Sci Rep. 2023 Apr 12;13:6017. doi: 10.1038/s41598-023-32674-6 (PMC10097698; doi:10.1038/s41598-023-32674-6)
Supplement: Supplementary file 5 — Supplementary Information 5. [file 41598_2023_32674_MOESM5_ESM.docx]

**Additional file 5: Statistics**

P-values from the multiple comparison tests (Table 1 and Table 2)

| **Comparison** | **Time** | **SD vs. FFC** | **SD vs.**  **FFC-DIA** | **SD vs.**  **FFC-DIA-S** | **FFC vs.**  **FFC-DIA** | **FFC vs. FFC-DIA-S** | **FFC-DIA vs. FFC-DIA-S** |
| --- | --- | --- | --- | --- | --- | --- | --- |
| Bodyweight  (kg) | T1 | <0.0012 | 0.0036 | 0.0036 | <0.0012 | <0.0012 | ns |
|  | T2 | <0.0012 | <0.0012 | <0.0012 | 0.0036 | 0.0072 | ns |
| Total Body Fat (%) | T1 | <0.0012 | <0.0012 | <0.0012 | ns | ns | ns |
|  | T2 | <0.0012 | <0.0012 | <0.0012 | ns | 0.023 | ns |
| Fat-free body mass (kg) | T1 | <0.0012 | ns | ns | 0.0012 | 0.0096 | ns |
|  | T2 | ns | ns | ns | ns | ns | ns |
| Systolic BP (mmHg) | T1 | ns | ns | ns | ns | ns | ns |
|  | T2 | ns | ns | ns | ns | ns | ns |
| Diastolic BP (mmHg) | T1 | ns | ns | ns | ns | ns | ns |
|  | T2 | ns | ns | ns | ns | ns | ns |
| Mean BP (mmHg) | T1 | ns | ns | ns | ns | ns | ns |
|  | T2 | ns | ns | ns | ns | ns | ns |
| Mean HR (beats/min) | T1 | ns | <0.0006 | <0.0006 | <0.0006 | 0.038 | ns |
|  | T2 |  |  |  |  |  |  |
| **Circulating metabolic biomarkers** | | | | | | | |
| Plasma glucose (mM) | T1 | ns | <0.0006 | <0.0006 | <0.0006 | <0.0006 | ns |
|  | T2 |  |  |  |  |  |  |
| Plasma fructosamine  (µM) | T1 | ns | <0.0006 | <0.0006 | <0.0006 | <0.0006 | ns |
|  | T2 |  |  |  |  |  |  |
| Plasma TG (mM) | T1 | 0.042 | 0.0092 | 0.037 | ns | ns | ns |
|  | T2 | 0.0030 | 0.0084 | ns | ns | ns | ns |
| Plasma TC (mM) | T1 | 0.0006 | 0.0012 | 0.0036 | ns | ns | ns |
|  | T2 | 0.0024 | 0.0090 | 0.013 | ns | ns | ns |
| Plasma insulin (pM) | T1 | ns | 0.013 | 0.0054 | <0.0006 | <0.0006 | ns |
|  | T2 |  |  |  |  |  |  |
| Plasma glucagon (pM) | T1 | <0.0006 | <0.0006 | <0.0006 | <0.0006 | <0.0006 | ns |
|  | T2 |  |  |  |  |  |  |
| **Circulating kidney biomarkers** | | | | | | | |
| Plasma creatinine  (mM) | T1 | 0.0042 | <0.0006 | <0.0006 | <0.0006 | 0.044 | ns |
|  | T2 |  |  |  |  |  |  |
| Plasma urea (mM) | T1 | ns | <0.0006 | <0.0006 | 0.018 | 0.017 | ns |
|  | T2 |  |  |  |  |  |  |
| Plasma NGAL (ng/mL) | T1 | ns | ns | ns | ns | ns | ns |
|  | T2 |  |  |  |  |  |  |
| **Urinary kidney biomarkers** | | | | | | | |
| Urinary glucose (mM) | T1 | ns | <0.0006 | <0.0006 | <0.0006 | <0.0006 | ns |
|  | T2 |  |  |  |  |  |  |
| UACR (mg/g) | T1 | ns | ns | ns | ns | ns | ns |
|  | T2 |  |  |  |  |  |  |
| UPCR (mg/g) | T1 | ns | <0.0006 | ns | <0.0006 | ns | 0.012 |
|  | T2 |  |  |  |  |  |  |
| UNGALCR (ng/mg) | T1 | ns | <0.0006 | <0.0006 | <0.0006 | 0.044 | ns |
|  | T2 |  |  |  |  |  |  |
| **Functional kidney parameters** | | | | | | | |
| GFR (ml/min/pig) | T1 | <0.0012 | ns | ns | 0.02 | ns | ns |
|  | T2 | 0.0024 | <0.0012 | 0.014 | ns | ns | ns |
| Resitive index | T1 | 0.0042 | 0.012 | <0.0006 | ns | ns | ns |
|  | T2 |  |  |  |  |  |  |
| **Kidney morphology** | | | | | | | |
| Kidney weight (g) | T2 | 0.0042 | <0.0006 | 0.0054 | ns | ns | ns |
| Kidney fibrosis | T2 | ns | ns | ns | ns | ns | ns |
| Average ME score (0-3) | T2 | NA | NA | NA | ns | ns | ns |
| Glomerulus area (µm^2^) | T2 | 0.0018 | 0.0090 | 0.0090 | ns | ns | ns |

Average ME score: SD not included in the group-wise comparison since all SD had score 0
