# Supplementary material for: Functional and morphological renal changes in a Göttingen Minipig model of obesity-related and diabetic nephropathy
Source: Sci Rep. 2023 Apr 12;13:6017. doi: 10.1038/s41598-023-32674-6 (PMC10097698; doi:10.1038/s41598-023-32674-6)
Supplement: Supplementary file 6 — Supplementary Information 6. [file 41598_2023_32674_MOESM6_ESM.docx]

**Additional file 6: Haematology and clinical chemistry parameters**

Haematology and clinical chemistry parameters of castrated male Göttingen Minipigs diet-fed for approx. 1 year on standard diet (SD) or fat, fructose and cholesterol rich diet (FFC) with or without additional salt (S) and with or without streptozotocin-induced diabetes (DIA).

| **Parameter** | **SD**  n=8 | **FFC**  n=16 | **FFC-DIA**  n=12 | **FFC-DIA+S**  n=9 | **Overall *P*-value** |
| --- | --- | --- | --- | --- | --- |
| Leukocyte | 9.46 (4.32-10.23)^7^ | 9.81 (7.82-11.88)^13^ | 9.82 (6.65-10.74)^8^ | 11.36 (8.57-14.52)^8^ | NS |
| Erytrocyte | 6.34 (5.88-7.53)^7^ | 6.55 (5.74-7.05)^13^ | 5.57 (5.32-7.87)^8^ | 5.60 (4.72-6.52)^8^ | ^#^NS |
| Haemoglobin | 6.8 (5.9-8.1)^7^ | 6.8 (6.1-7.3)^13^ | 6.3 (5.5-7.6)^8^ | 6.0 (5.4-6.6)^8^ | NS |
| Haematocrite | 0.34 (0.30-0.40)^7^ | 0.33 (0.32-0.36)^13^ | 0.30 (0.28-0.39)^8^ | 0.30 (0.27-0.34)^8^ | NS |
| Platelets | 493 (340-560)^7^ | 490 (427-561)^13^ | 844 (566-1032)^8^ | 775 (268-975)^8^ | NS |
| P | 1.87 (1.79-2.07)^7^ | 2.11 (2.00-2.27)^14^ | 2.01 (1.84-2.11)^8^ | 2.06 (2.02 – 2.20)^8^ | NS |
| Ca | 2.44 (2.39-2.58)^7^ | 2.53 (2.43-2.57)^14^ | 2.45 (2.43-2.45)^8^ | 2.41 (2.34-2.48)^8^ | NS |
| Ma | 0.98 (0.87-1.01)^7^ | 0.93 (0.88-0.98)^14^ | 0.97 (0.94-1.00)^8^ | 0.96 (0.90-0.99)^8^ | NS |
| Na | 142.5 (141.4-144.5)^5^ | 143.6 (142.4-144.0)^13^ | 136.4 (135.7-137.1)^7^ | 135.6 (133.8-138.4)^7^ | SD,FFC>FFC-DIA,FC-DIA+S (*P*<0.0001) |
| K | 4.36 (4.21-4.48)^5^ | 4.42 (4.07-4.55)^13^ | 4.56 (4.33-4.72)^7^ | 4.59 (4.36-4.80)^7^ | NS |

#Logaritmic transformation
