# Supplementary material for: Functional and morphological renal changes in a Göttingen Minipig model of obesity-related and diabetic nephropathy
Source: Sci Rep. 2023 Apr 12;13:6017. doi: 10.1038/s41598-023-32674-6 (PMC10097698; doi:10.1038/s41598-023-32674-6)
Supplement: Supplementary file 7 — Supplementary Information 7. [file 41598_2023_32674_MOESM7_ESM.docx]

**Additional file 7: 24 hour systemic blood pressure and heart rate.**

Heart rate (A, B), systolic blood pressure (C, D), diastolic blood pressure (E, F) and mean blood pressure (G, H) in 48 intervals of 30 minutes each from 10:30 am to 10:30 am the following day. Male, castrated Göttingen Minipigs fed with standard diet (SD) or fat, fructose and cholesterol rich diet (FFC) with or without additional salt (S) and with or without streptozotocin-induced diabetes (DIA). Left panel: T1, right panel: T2. Blue shaded area is the Day-time period and grey shaded area is the Night-time period used in the statistical analysis. Median±interquartile range, n=6-14.
