# Supplementary material for: Functional and morphological renal changes in a Göttingen Minipig model of obesity-related and diabetic nephropathy
Source: Sci Rep. 2023 Apr 12;13:6017. doi: 10.1038/s41598-023-32674-6 (PMC10097698; doi:10.1038/s41598-023-32674-6)
Supplement: Supplementary file 8 — Supplementary Information 8. [file 41598_2023_32674_MOESM8_ESM.docx]

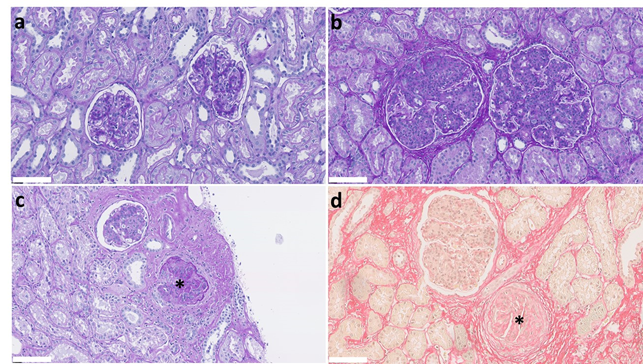


**Additional file 8: Histopatholgical findings**

Examples of histopathological findings observed in animals from the FFC, FFC-DIA and FFS-DIA+S groups. Compared to the SD group (a), animals from the FFC-DIA group (b-d) showed enlarged glomeruli with mesangial expansion, increased glomerular size and hyperplasia of the glomerular cells (b). In few cases glomerulosclerosis (asterisk, c and d) and extensive periglomerular fibrosis was observed (c and d). a, b and c: Periodic acid Schiffs (PAS), d: picrosirius red (PSR) staining.
